# Cancer as the “perfect storm”? A qualitative study of public attitudes to health conditions

**DOI:** 10.1002/hsr2.16

**Published:** 2017-10-27

**Authors:** Liz Morrell, Suzanne Sayuri Ii, Sarah Wordsworth, Roger Wilson, Sian Rees, Richard Barker

**Affiliations:** ^1^ Oxford‐UCL Centre for the Advancement of Sustainable Medical Innovation (CASMI), Radcliffe Department of Medicine University of Oxford Oxford UK; ^2^ Health Economics Research Centre, Nuffield Department of Population Health University of Oxford Oxford UK; ^3^ Oxford NIHR Biomedical Research Centre University of Oxford Oxford UK; ^4^ National Cancer Research Institute Consumer Forum London UK; ^5^ Health Experiences Institute, Nuffield Department of Primary Care Health Sciences University of Oxford Oxford UK

**Keywords:** public attitudes, qualitative, hope, fear, cancer, dementia

## Abstract

**Aims:**

Our aim is to identify important attributes of major diseases that shape how they are perceived by the public.

**Methods and Results:**

Four focus groups among members of the public were recruited, in March and October 2016, and used semistructured discussion to explore important attributes of cancer, heart disease, stroke, dementia, mental illness, and infectious disease. Common themes were identified by using inductive thematic analysis.

Five themes were identified: fear, impact on family and friends, hope, detection, and prevention. Fear of cancer includes not only fear of death but also of aggressive treatments. Loss of dignity is feared in dementia, while infectious disease raises fear of uncontrollable “plague”; in contrast, people with mental illness may themselves be seen as a potential threat. The impact of cancer and its treatment on family and friends was described as intense and all‐consuming, even for those not involved directly in caring; with dementia and stroke, the family impact is taking on care, including funding, over the long term with little expectation of improvement. Hope is a major theme in cancer and stroke recovery, linked with the need to take action, often expressed in aggressive language of “fighting,” but seen as futile in dementia. Detection difficulties for “silent” cancers mean that real treatment opportunities are missed; cardiovascular and infection risk, however, are seen as easy to identify and act on, whereas mental illness and dementia are seen as poorly diagnosed and with limited treatment options. Prevention awareness is high for cardiovascular disease and infection, lower for cancer, and limited for dementia and mental health.

**Conclusion:**

Although themes overlap across diseases, the specific concerns are different and each condition has a unique profile. Quantifying the relative importance of these themes could allow their incorporation in decision‐making, not only when they occur as a named disease but also in any relevant condition.

## Abstract 2

This qualitative study aimed to identify important attributes of major illnesses that shape how they are perceived by the public. Our results identified 5 common themes: fear, impact on family and friends, hope, detection, and prevention. Although the themes overlapped across the 6 health conditions discussed, the specific concerns differed, with each condition showing a unique profile and pattern of overlaps with the others.

## 1 INTRODUCTION

Many countries face the issue of setting health care priorities when the resources available are insufficient to provide all the health care that society demands. In a publicly financed health care system, members of the public fund health‐care provision through their taxes and are the beneficiaries of that care. It is therefore important that public views inform prioritization policies.^1^ Those public views are based not only on rational evaluation of evidence but also on experience and anecdote that create individuals' subjective views, and the challenge for policy‐makers is to incorporate an appropriate balance of both aspects.

Cancer presents an interesting case. Cancer is generally a feared disease 2, 3, 4; euphemisms such as “the Big C” remain common, and decisions not to fund new cancer drugs have attracted media focus and strong public reaction.^5^, ^6^, ^7^, ^8^ Cancer also receives special treatment in health funding policy: for example, the Cancer Drugs Fund in England is a ring‐fenced fund for cancer drugs, established in 2010 based on the belief that “*it is possible that society values benefits to patients with cancer more highly*, *all else being equal*, *than benefits to patients suffering other conditions*.”^9^ However, in a recent literature review, we found that when studies ask people to trade off funding cancer care against equivalent health improvement for other conditions, they do not consistently show a preference for cancer funding.^10^ For example, a large‐scale choice study that addresses this question directly for the UK found no support for prioritizing cancer above other conditions all else being equal but a preference for obtaining greater health improvement overall.^11^

Other prioritizing factors have been evaluated in similar trade‐off studies. There is a body of evidence supporting prioritizing of severe disease, although the definitions of severity vary^10^, ^12^, ^13^; severity is not explicitly prioritized in the UK, although National Institute for Health and Clinical Excellence (which makes funding recommendations for England's National Health Service) can give special consideration to severe disease.^14^ Short life expectancy and rarity are given funding priority in the UK, through policies such as National Institute for Health and Clinical Excellence's end‐of‐life criteria,^15^ Highly Specialised Technologies appraisal proces,^16^ and the Scottish Medicines Consortium (SMC)'s end‐of‐life, orphan, and ultra‐orphan processes^17^; the evidence on preference is mixed^10^, ^11^, ^13^, ^18^ with an important UK study showing no preference for either.^11^ However, encouraging orphan drug development is consistent with an egalitarian preference (providing “something for everybody”) seen in some studies,^19^, ^20^ and both short life expectancy and the rare metabolic disease of childhood seen by the Highly Specialised Technologies process would likely be considered “severe” by most observers.

Hence, across a range of prioritizing factors, the preference literature is not entirely consistent with the observed public and policy response, suggesting that the (tangible) concept of maximizing health improvement might not capture (perhaps subjective) broader aspects that are important to the public. Cancer is not, of course, the only illness of public concern, although it is the only named disease to be enshrined in a specific funding policy: ischemic heart disease was the leading cause of death in the United Kingdom until 2015 when it was supplanted by dementia and Alzheimer disease^21^; recent years have seen the concerns with global viral epidemics and antibiotic resistance,^22^ and mental health‐care resourcing is the subject of current debate.^23^ Advocates for such conditions would also argue that the specifics of the condition are not fully accounted for in funding decisions; perhaps cancer has a particular combination of attributes—the “perfect storm”—which explains its predominance in public attitudes and policy. If such valued attributes can be identified across diseases, they could be explicitly in prioritization decisions, not only when they occur as a named condition but in any other condition where they might apply.

This paper reports qualitative research undertaken with members of the public in the United Kingdom, with the objective of identifying important attributes of major diseases that shape how they are perceived by the public and which may not be explicitly accounted for in health technology appraisals and cost‐effectiveness analyses. This work is timely as the English Cancer Drugs Fund recently underwent reform while retaining ring‐fenced funding^24^^,^ 25 and there are calls for reform of health technology appraisal processes from UK stakeholders.^26^, ^27^

## 2 METHODS

Field researchers were LM and SSI, employed by the Centre for Advancement of Sustainable Medical Innovation, a policy research group at the University of Oxford. LM is a health economist, SSI is a social scientist, and both have experience in mixed method research. LM is a trained moderator. The authors include a cancer patient advocate (RW), who was involved in initial study design and provided critical input to the interim summaries and drafts of the manuscript.

Thirty participants in total (4 focus groups) were recruited from 2 sources, to provide a range of perspectives. Fifteen participants (2 focus groups, n = 7 and n = 8) were recruited via the email bulletin of Healthwatch Oxfordshire (a health and social care consumer watchdog); recipients of the bulletin have signed up because they have a particular interest in health care. Interested respondents emailed LM directly. Fifteen further participants (2 focus groups, n = 8 and n = 7) were recruited through a commercial recruiter in the High Wycombe area (Leo House Fieldwork, UK), from a telephone panel representative of the UK general public; no participant contact details were provided to the researchers. The locations were chosen for convenience near to our university. For all groups, we excluded people with a (self‐reported) current diagnosis of any of the illnesses under discussion or recent (past 6 months) experience with a close family member; this exclusion was to avoid possible distress to participants and to reduce the risk of a current “patient” perspective dominating the discussion of general public views. The 2 sets of groups are referred to here as “health‐engaged” (HE) and “representative public” (RP).

The focus groups were run in local conference facilities in March (HE) and October (RP) 2016 and were moderated by LM with SSI observing and taking notes. Each group was 90 minutes long and explored perceptions of 6 categories of illnesses: cancer, heart disease, stroke, dementia, mental illness, and infectious disease. These were chosen from a list elicited through discussions among the authors (Appendix 1) and selected according to the following criteria: (i) sufficiently prevalent to be of concern to a general audience; (ii) a mix of life‐threatening and chronic conditions; (iii) some which affect children; and (iv) excluding conditions that might be seen as largely self‐induced (eg, obesity), where we were concerned this belief might dominate the discussion.

The participants were told that the researchers were from a health policy research group at the University of Oxford, working on a project aiming to explore how the National Health Service can best deliver care that is important to the public. Before the groups, the HE participants were asked to respond by email to a question: *thinking about illnesses or health conditions that could affect you personally*, *what would you say is your greatest concern?* A word cloud was created from the responses (Appendix 2) and used as a stimulus in all focus groups to initiate discussion, which then followed a semistructured discussion guide (Appendix 3) to discuss each of the health conditions in turn.

The groups were audio‐recorded, transcribed verbatim (UK Transcription Ltd, UK), and analyzed for common themes using a framework approach.^28^ A coding frame was derived inductively,^29^ starting from an outline structure based on the 6 health conditions; all focus groups were coded according to this common structure, using MAXQDA software v12.2.0 (VERBI, Germany). Overarching themes across all 4 groups were then constructed by consensus, by combining codes with related content from the 6 disease areas, regardless of the initial derivation and naming of the code. LM and SSI each coded a subset of the transcripts and analyzed a subset of themes across disease areas, with the HE and RP participant types being analyzed separately; both reviewed each other's work, with differences resolved by discussion. Each researcher summarized findings for 1 participant type, and these interim summaries were reviewed by all authors. The HE summary was sent to the participants as a check that we had accurately reflected their views; most agreed with the summary, with a specific point of accuracy from 1 respondent, which we corrected. The analyses were synthesized by LM to provide an overall perspective, with divergences between the participant types identified where they occurred.

Ethics approval was granted by the University of Oxford Medical Sciences Interdivisional Research Ethics Committee (R44122/RE002). All participants provided written informed consent before participating. Quotes are anonymized by using participant number; gender (M/F) and group type (HE/RP) are indicated. Reporting is consistent with COREQ (consolidated criteria for reporting qualitative research).^30^

## 3 RESULTS

The HE participants were largely retired, with an age range of 50 to 85. The groups were both mixed gender (total 9 male, 6 female), and all but 1 participant were of white British or European ethnicity. They were actively engaged in health and health care (for example: members of a general practice or hospital patient participation group, involved in clinical trials, and worked in public health), had experience with the relevant health conditions either personally (for example: a cancer treated surgically and mild anxiety diagnosed some years ago) or through close family and friends, and used health information from a range of resources. The RP participants were younger (range 35‐71), mostly working and/or caring for children, typically had less direct experience and knowledge about health and health care, and were largely reliant on the media for health information, for example, television advertising campaigns. The groups were both balanced for gender (total 7 male, 8 female), and all but 1 participant were of white British or European ethnicity.

Our analysis identified 5 common themes; in some cases, these were derived directly from the codes (for example: “family and friends” was a code for all illnesses) but in others were built up from codes containing related concepts (for example: “detection” was derived from codes including “diagnosis,” “onset,” and “early intervention”). Findings are presented by theme, contrasting the specific areas of concern for the illnesses considered, and among the 2 types of respondents; the order of themes as presented reflects the general flow of the semistructured group discussions.

### 3.1 Fear

#### 3.1.1 Cancer

Fear and dread was an immediate response. “*I feel the very word is an immediate thing*. *If it is said to you it strikes terror*” (6: F, HE). The basis of the fear was twofold. First, fear of death: “*From the time of diagnosis you are going towards your end*” (2: M, HE). This is despite acknowledging that cancer mortality rates have reduced because the participants did not believe that they will necessarily be the one to benefit: “*Even if they said to you*, ‘*We have caught it early and there is a good percent*.’ *I just know personally that I would be thinking*, ‘*With my luck I am that 20%*.’ *Even if they tell me I have got an 80% chance of surviving it*.” (23: F, RP).

Second, fear of the treatments: specifically, the side effects of drugs, the duration of treatment path, and body disfigurement due to surgery. The respondents commented on low quality of life during cancer treatment, which is particularly distressing in a life already shortened by the illness, and although some respondents cited inspirational examples of cancer survivors, the fear of death despite treatment remained “*The appalling consequences of these treatments and then nothing at the end except death*” (15: F, HE). Fear of death was the stronger emotion in the RP group, with both aspects feared in the HE groups. One HE respondent had concluded that she would not accept chemotherapy if she was diagnosed with cancer, instead choosing to minimize her risk by eradicating “*toxins*” from her life; this was met with some incredulity from the others, none of whom explicitly agreed with her choice, but there was broad agreement with the premise of unpleasant treatments.

For some respondents, past experience of cancer and its treatment had reduced the dread: “*you're a member of a club*, *you can walk down that* [*oncology*] *corridor*” (9: M, HE), as had more openness in society generally to discuss cancer. However, for most, dread of cancer was intensified by experience, observing or supporting a family member or friend with cancer, and messages about prevalence: “*there is an advert* […] *on the telly at the moment saying that one in two of us will be affected by cancer*. *I just sat and looked at my husband*, ‘*Well that's one of us*’” (23: F, RP). The respondents described how former patients experience heightened awareness of their own risk and increased sensitivity to possible symptoms: “*My wife had a mastectomy 42 years ago*. … *Any time she feels a little bit ill about something*, *she begins to worry*” (8: M, HE).

#### 3.1.2 Heart disease

Heart disease did not create such strong emotions of fear even among those with risk factors: “*It is not the same death sentence* [*as cancer*]. *There is not a timetable towards the end*, *no*” (2: M HE). The participants believed that risk factors were known and readily observed (such as blood pressure, diet, and activity) and that they could choose to take action to reduce their risk: treatments were also acknowledged to be good, notably in contrast with cancer: “*Cardiac medicine* […] *is fantastic*. *You've got a very good chance of surviving it*, *and the treatments are not horrible and aggressive as they are for cancer treatments*” (11: F, HE).

#### 3.1.3 *Strok*e

The fear was not of a shortened life but potentially a long life with a loss of independence. Treatments in themselves were not feared—in fact were acknowledged to be good. The participants were aware that outcome was dependent on the speed of accessing treatment: “*The dread is if there is a gap* [*to treatment*], *you could be left more disabled*” (13: F, HE).

#### 3.1.4 Dementia

The main fear was of loss of self and dignity, particularly during the process of degeneration when the person was aware of what was happening, and in the RP groups specifically, this led to discussions of assisted suicide. However, most participants believed that patients with advanced dementia are not sufficiently aware to be distressed by their condition: “*Once you're down that road*, *and you're beyond fear or hope or anything*, *but if you're getting to a point where you think*, ‘*This isn't normal memory loss*, *there's something wrong with me*,’ *that must be terrifying*.” (11: F, HE).

#### 3.1.5 Mental illness

Fear was less about being diagnosed themselves, but fear of others: fear of not responding appropriately to someone with a mental health condition, and for their own safety around such people: “*You are frightened that that person is a threat to you as well*” [condition not named] (7: F, HE). The RP groups in particular were concerned that people who were considered a danger to themselves or the public should have appropriate care, secure where necessary. Usage of names of specific mental health conditions was limited; the respondents used the terms *anxiety* and *depression* for their own or others' known diagnosis, but examples of observed or reported behavior were generally unnamed.

#### 3.1.6 Infectious diseases

The HE group discussed fear of “plague,” where the spread cannot be controlled. Diseases with no immediate personal risk evoked less fear, even if they were untreatable (eg, Ebola). In contrast, infectious diseases did not create fear in the RP groups, in the belief that this country is not affected, and we are able to protect ourselves. Antibiotic resistance was raised as a concern, particularly in the HE groups: “*We have infectious diseases under control at the moment but there is every possibility that that will cease to be the case over the next decade and I find that a bit scary*.” (2: M, HE).

### 3.2 Impact on family and friends

#### 3.2.1 Cancer

The participants described the intense, “*all‐consuming*” (15: F, HE) effect of cancer and its treatment, on the family. The participants with direct experience described anxiety, powerlessness, the way their life was “*on hold*” (15: F, HE) throughout treatment, and guilt if they gave way to their own emotions or needs: “*You are the support network and you feel helpless*, *what can you do*?” (20: F, RP). Family and other carers are left with a fear of following the same path and extreme awareness of symptoms that could be early indicators of cancer in themselves; these sensitivities, as with former patients, persist over a long term. “*My children are in dread of bowel cancer*, *because they saw their father* … *and it has affected them quite markedly*” (15: F, HE).

Notably in cancer, the participants also described the impact of cancer outside their immediate family; most could give examples of friends being diagnosed and undergoing treatment and had been emotionally affected, even if the respondent had not been involved directly: “*A friend of mine was admitted to hospital two weeks ago with cancer of the pancreas*. *He is in immense pain*. *I have not seen him because he is 200 miles away*. *That affects me more at the moment than anything else*.” (1: M, HE).

#### 3.2.2 Heart disease

The participants agreed that they would care for a relative who needed help, but the discussion did not have the emotional intensity seen for cancer, stroke, or dementia.

#### 3.2.3 Stroke and dementia

The participants described the role of carer that families have to take on, which may include funding high costs for care, or adjustments to housing to take account of disability. The need for care is seen as long term, with little or no expectation of improvement in dementia or from major disability post stroke: [dementia] “*There is no end in sight*. *It just goes on and on and on*” (5: M, HE). Dementia was seen as particularly devastating for the relationships, such as the spouse or offspring becoming a carer: “[*dementia*] *is devastating on those nearest*, *the family and friends and the ones who are really near to them*. *It destroys them*” (3: F, HE).

#### 3.2.4 Mental illness

In the HE groups, the main comments came from 1 participant describing his difficulties getting a diagnosis and treatment for his daughter. The RP groups predominantly discussed the long‐term impact on children, due to the behavior of a parent: “*But it* [*children's recovery*] *took a long time*, *it took years*” (18: M, RP).

### 3.3 Hope, fight, and taking action

#### 3.3.1 Cancer

Hope was an important concept among the HE group, as a necessary response to cancer; the participants described hope as largely coming from within, as an attitude of mind dependent on personality and the individual's will to live. However, some in the RP group did not associate hope with cancer (“*Hope*? *No*, *not at all*.” [27: F, RP]) or saw hope as passive, preferring to rely on medicine: “*I'd rather invest my time in technology*, *rather than hope*” (29: M, RP).

Whether or not it was expressed in hope, there was a common preference for taking action and hence retaking control. The participants described the aims of taking action as a cure, longer life, and a return to normality—these contrast with perceptions of dementia: “*Because with cancer if you fight it and you win you then have a normal life again*” (23: F, RP). Action could include alternative therapies or lifestyle changes, particularly if drug options had been exhausted.

Discussions of taking action in cancer used aggressive vocabulary such as “fight” and “battle.” “*If I was diagnosed tomorrow*, *I would think*, ‘*Right*, *we've got a fight on our hands*,’ *rather than*, ‘*Oh*, *that's it*.’” (28: M, RP). Specifically in the RP groups, the participants personified cancer as a vindictive entity to fight against: “*Once you have been diagnosed with cancer it comes back and it comes back and it comes back until it gets you*” (23: F, RP). The necessity of fighting and being positive was expressed strongly in all groups; in one RP group, most participants reacted strongly against one member who they thought was not taking a sufficiently positive attitude. A minority expressed fear that you might not win, resulting in a sense of failure: “*Some people would say the psychological thing was then you are disappointing*. *You fought it but you failed*.” (7: F, HE).

#### 3.3.2 Stroke

Hope and fighting were mentioned by some respondents in the context of recovery from stroke‐related disability: “*If you've been paralysed*, *you try and fight your way back to health*” (14: M, HE).

#### 3.3.3 Dementia

There was little focus on hope or fight because of the perceived lack of effective treatment or opportunity to reverse the condition: “*You're never going to get your life back*” (22: F, RP). Any “fighting” was expected to be by the family, fighting to get quality care and services. “*My sister*… *spent a lot of time doing lots and lots of research*. *She was doing the fighting that mum couldn't do*.” (29: M, RP).

#### 3.3.4 Heart disease and mental illness

Hope was not raised spontaneously as a relevant concept in these conditions, in contrast to arising naturally for the others; due to time constraints, this was not probed extensively in the groups. In the case of mental illness, only 1 respondent directly talked about a colleague “*fighting it* [*depression*]” (25: M, RP); discussion of action focused on pushing for a diagnosis and suitable treatment, similarly to dementia. For heart disease, hope was seen as less relevant because of good treatment options and well‐known management strategies: “*There is more one could do about heart disease than you can do about cancer*. *You can adjust your lifestyle*” (2: M, HE); notably, the actions that could be taken were not described in fighting terms in these groups.

#### 3.3.5 Infectious disease

Hope was reflected as control, via being prepared or preventing transmission (HE groups).

### 3.4 Detection

#### 3.4.1 Cancer

The participants agreed on the importance for prognosis of early detection and made frequent references to cancers caught too late for effective treatment. The invisibility of some cancers contributed to fear of the disease, in contrast to heart disease where the risk factors were considered to be well known. Reasons for late detection included “silent” cancers that are painless or symptomless (pancreatic and ovarian cancers) and misdiagnosis where a cancer occurs in an unexpected population or is mistaken for a less serious illness such as a “*stomach bug*” (1: M, HE): “*When the young girl goes and is told*, ‘*It'll be fine*. *It's just that you're young*, *your periods are going up the shoot*’ … *It's then when people are fooled into* [*thinking*] *it will pass*.” (13: F, HE).

The participants agreed that significant progress had been made in raising awareness of what signs to look for and availability and acceptance of screening: “*Breast screening was probably something that wasn't spoken about a few generations ago but is now perfectly acceptable*. *Even the bowel screening test you can do in your own bathroom and send it in the post*.” (5: M, HE).

#### 3.4.2 Heart disease and stroke

In contrast to cancer, risk factors were thought to be well understood and readily detected. Particularly in heart disease, detection of people at risk was seen as straightforward including lifestyle and simple blood pressure monitoring, and ways to reduce risk were very familiar: “*the heart part of it is they have a better chance because of the screening*. *It can be picked up*. *With the cancer it is picked up when it is probably too late*” (3: F, HE).

Discussion of risk factors was integrated with early intervention and prevention, in contrast to the other conditions. The participants were also aware of educational campaigns to help people recognize a stroke and take action; however, in the RP groups, there was some lack of understanding of the difference between heart disease and stroke: “*I didn't know until a few years ago what the difference was*. *I thought it was all the same thing*.” (29: M, RP).

#### 3.4.3 Dementia and mental illness

In both cases, the participants commented on the difficulties of detection and diagnosis, with mental illness in particular felt to cover a broad spectrum of conditions that are largely poorly understood and hard to diagnose correctly. The participants gave examples of incorrect diagnoses, inappropriate support, and the role of family and friends in getting help and were concerned that because of their condition, patients may be unable to able to communicate effectively with health professionals and carers. Notably, in the RP groups, there was disagreement over the distinction between some forms of mental illness (named as schizophrenia by 1 group) and criminality and when a secure care environment was appropriate: “*I think what you are getting at is there might be two people doing exactly the same things*, *but one is mentally ill and one isn't*.” (16: M, RP).

### 3.5 Causes and prevention

#### 3.5.1 Cancer

Genetics was seen as a key causative risk, for which little prevention was possible, beyond a generally healthy lifestyle, and at the extreme, preemptive surgery. Specific cancers were identified as having known causes; the most notable were smoking (lung cancer) and sun exposure (skin cancer). The HE groups reported taking more proactive steps to avoid cancer: “*Cancer is in my family on all sides*, *so I expect I might eventually get it*. *I don't know*. *But I make sure I eat well*. *And we exercise*” (13: F, HE). In contrast, the RP groups considered cancer risk to be fairly random and dependent on individual triggers: “*That is cancer isn't it*, *cancer is a lottery*” (23: F, RP) and reported few specific preventive behaviors.

#### 3.5.2 Heart disease and stroke

In contrast to cancer, there was high awareness of the role of lifestyle and that well‐known changes could affect risk at any stage: “*More people diagnosed with a heart condition would feel that they can and must do something about it*” (4: M, HE). The participants were generally aware of their own risk level. Family history, a health scare, or diagnosis of a heart condition or other chronic condition typically acted as a stimulus for lifestyle change and uptake of screening: “*The writing was on the wall and I made sure that I was doing everything I could to prevent a recurrence*” (9: M, HE). In the RP groups, many had given up smoking for predominantly financial and social taboo reasons.

#### 3.5.3 Dementia

For dementia, there was little awareness of specific causes, beyond the aging process and an element of luck. Some participants had strong family histories of dementia and believed they could be at higher risk: “*Am I going to be more prone to it*? *There is always that thought in the back of my mind that I may go down that way*.” (4: M, HE).

#### 3.5.4 Mental illness

The participants in these groups had little to say on causes or their prevention and did not volunteer their personal risk level.

#### 3.5.5 Infectious disease

These were believed to be more manageable, even when incurable, because there is a target infectious agent; transmission or progression (eg, HIV/AIDS) can be controlled: “*You would generally feel that there was a better prospect of early detection and of being able to manage it* … *because there is a discernible cause for an infectious disease*” (2: M, HE). The participants agreed that individuals in an affluent society can affect their own risk of infection in a range of circumstances (eg, vaccination, mosquito protection, and avoiding body contact) and generally have a choice whether to visit an infected area.

## 4 DISCUSSION

This work aimed to identify important attributes of major illnesses that shape how they are perceived by the public. To our knowledge, it is the only study to provide a direct comparison between perceptions of significant health conditions in the United Kingdom. Our results identified common themes across conditions, but the specific concerns differed, with each condition showing a unique profile and pattern of overlaps with the others.

Alongside fear of death, significant elements in fear of cancer are the aggressive nature of treatments and their impact on the patient and family. In contrast, loss of dignity is feared in dementia, while infectious disease raised fear of uncontrollable plague; people with mental illness may themselves be seen as a potential threat. Hope is a major theme in cancer and in stroke recovery, linked with the need to take action, often expressed by using personification and aggressive language of “fighting”; this is seen as futile in dementia, with family “fighting” for support for dementia and mental health patients. A unique feature of cancer in this study was the pairing of treatment availability with the potential for late or misdiagnosis, meaning real treatment opportunities can be missed with a negative impact on outcomes. Cardiovascular and infection risks, however, are seen as easy to identify and act on, whereas mental illness and dementia are seen as poorly diagnosed but with limited treatment options. Awareness and engagement with prevention activities were high for heart disease and stroke, lower for cancer beyond specific known causes, and limited for dementia; prevention was not discussed for mental health, possibly due to low awareness of causes, and although awareness was high, infection prevention was largely not seen as an issue in a developed country with modern health care.

The themes we identified are consistent with the findings of a recent literature review of the elements of cancer fear,^31^ which also notes the use of “battle” metaphors and personification of the disease. The authors suggest that this can result in skepticism about preventability of cancer, leading to reduced preventative behaviors; similarly, a recent empirical study showed that “enemy” metaphors in cancer education reduced intentions for self‐restraining preventive behaviors.^32^ Fighting metaphors have also been criticized for communicating vulnerability or failure if the disease progresses^33^, ^34^ and contributing to overtreatment, particularly in the context of aggressive treatment and intense family engagement, making it difficult for a patient or clinician to step away from unwanted or futile treatment.^35^, ^36^ The fear of cancer treatments we observed may be the consequences of an aggressive chemotherapy paradigm. Similar observations on fighting metaphors can be found in the popular press.^37^ Alternatives to “war on cancer” vocabulary, such as “no patient left behind,” have been proposed.^36^

Our findings are also broadly consistent with policy initiatives related to additional sources of value not captured in standard measures of health used in funding decisions. The value‐based pricing initiative of 2010 proposed additional weighting factors for decision‐making and included severity and carer impact.^38^ The SMC's Patient and Clinician Engagement process provides a mechanism to identify additional aspects of value,^17^ and themes discussed have included hope, independence, and toxicity.^39^ A recent independent review of SMC funding decisions proposed development of a “basket” of measures, to include wider societal benefits.^40^ Our findings may also point to aspects of health care that matter to the public beyond health maximization. However, any attempt to integrate such features in decision‐making faces significant implementation challenges, including operationalizing these concepts (an issue faced by value‐based pricing^41^), avoiding double‐counting, and defining a cost‐effectiveness threshold in the resultant new definition of health.

The need to take action or “fight” observed in some conditions may be relevant to patient and public acceptance of stratified medicine. Stratification implies not giving a treatment to patients for whom it is deemed unlikely to be effective based on biomarkers. However, treatment may be fulfilling the need for action and providing a weapon with which to fight; value may lie in “having a next option.” Further work is needed to understand the social and equity implications of increased stratification of treatment access.

Stratification is also relevant to the concept of unpleasant or challenging treatment regimes. Some of the value of stratification may be in avoiding morbidity from multiple, perhaps marginally effective treatments. To recognize that value, it is essential to capture the full morbidity effects of the avoided treatments. The dread of treatments expressed here raises the question of whether current measures of health‐related quality of life fully reflect such treatment disutility, both during treatment and over the following months, and whether patients differ from the general public in their willingness to tolerate toxicity. Measurement of health‐related quality of life is also relevant to the dread of loss of dignity, expressed here for dementia, raising further challenges including the need for proxy evaluations.

This study is limited by its small size and geographic coverage. The HE group did not appear to be representative in health‐care knowledge; however, with increased access to information via the Internet, it may be that this population represents the direction of travel for society in general and will increasingly become the norm. Additionally, we have not spoken to people across the full range of health engagement; the RP respondents were sufficiently engaged to participate, and attitudes may differ among groups that are hard to reach in health‐care education and provision. Further work among that broader population would help to refine our findings. The findings are also limited by the choice of diseases discussed, which was constrained to allow sufficient discussion of each one in the timeframe of a group discussion. Further, this study takes the perspective of only 1 stakeholder group—that of the general public—and other stakeholders including clinicians, patients, and policy‐makers also have a role to play in health‐care decisions. Finally, as a qualitative study, this work provides no guidance on the relative importance of any of the aspects described; this would require further work to distil the themes into specific attributes, followed by quantitative studies such as discrete choice experiments.

In conclusion, our study identified a range of themes that may represent additional aspects of value in cancer and other diseases, beyond straightforward health maximization. Each health condition was found to have its own combination of specific concerns. Separating out the particular aspects of value, as opposed to the overall perception of a named disease, would enable such features to be developed for explicit use in decision‐making and applied to any condition where that feature is relevant.

## CONFLICTS OF INTEREST

SSI receives funding from Celgene Corporation (Summit, New Jersey, USA). SR has received fees for leadership training from Takeda (High Wycombe, UK). RB is a nonexecutive director of Celgene Corporation (Summit, New Jersey, USA) and chairman of Image Analysis (London, UK). LM, SW, and RW declare no conflict of interest.

## Supporting information

Data S1 Supporting information item
