# Supplementary material for: Cancer as the “perfect storm”? A qualitative study of public attitudes to health conditions
Source: Health Sci Rep. 2017 Oct 27;1(1):e16. doi: 10.1002/hsr2.16 (PMC6034427; doi:10.1002/hsr2.16)
Supplement: Supplementary file 1 — Data S1 Supporting information item [file HSR2-1-e16-s001.zip]

**Supporting Information**

**Appendix 2 – Questions in each domain of the question guideline**

Questions on information provided to patients include asking patients i) where they got information to help them make a decision about whether to have chemotherapy before surgery; ii) which of these information sources they found most useful; (iii) what exactly the information was that helped them make the decision; (iv) whether they felt they were given enough information to allow them to make a decision; (v) if they felt they were not given enough information, what other information they would like to have received; and (vi) how they would like information presented to them (written, face-to-face, online).

Questions regarding the decision making process and psychological concerns include asking patients i) who made the decision in the end; (ii) what was difficult about making the decision; (iii) how certain they were about the decision at the time when they made the decision; (iv) how certain they are now that they made the right decision; and (v) if their certainty has changed, why it has changed. Patients are further asked whether vi) they do or did worry that their cancer will get worse whilst having chemotherapy; (vii) what period during chemotherapy and surgery they found most difficult, mentally and physically; and (viii) whether they worry that their cancer will come back.

Questions regarding patients’ experiences with the decision aid include asking patients i) how much time they spent using the decision aid; (ii) whether it provided additional information to that provided by their health professionals; (iii) whether the information was relevant to their decision and in what way it was relevant/not relevant; (iv) how the information factored into their decision; (v) whether the information was trustworthy; (vi) whether the information was presented in a way that was easy to understand; (vii) whether they perceived the decision aid to be too long, about right or too short; (viii) whether the amount of information was too much, about right, or too little; (ix) whether the decision aid favoured NAST, was balanced or favoured surgery; (x) whether they have other comments on the decision aid.

Questions regarding other factors which might have influenced patients’ decision include asking patients whether and if so, how the following factors influenced their decision: i) having breast conserving surgery (lumpectomy); (ii) being able to know whether the cancer responded to chemotherapy; (iii) having treatment sooner for the whole body, not just for the breast; (iv) being involved in a clinical trial (and whether their doctor talked to them about this); (v) their ability to have children in the future. Patients are further asked whether (vi) they are aware that breast cancer can be inherited in the family and whether that was relevant to their decision; (vii) what other issues they considered when making the decision, such as financial or logistic issues; and (viii) whether they have considered having a breast reconstruction.

**Appendix 3 – One page of the decision aid, describing a step-by-step approach for how to arrive at a treatment decision**

*Arriving at a treatment decision*

The previous pages have outlined the main options available to you now. The following steps may help you to make a decision whether or not to have chemotherapy or hormonal therapy before surgery. The decision making process may be easier if you follow these seven steps:

1. Understand your diagnosis and your risk of breast cancer recurring (coming back) as fully as you can.
2. Understand your options for treatment and the risks and benefits of these options.
3. Review the pros and cons of those options.
4. Assess the importance to you of the pros and cons.
5. If you are offered neoadjuvant treatment through a clinical trial, prioritise the pros and cons of the trial for you (and your family).
6. Get more information from your doctor or breast care nurse if you are unsure of anything or have more questions.
7. Discuss your preferred treatment option with your surgeon, medical oncologist, family doctor, your family and other significant people in your life.

You have already gone through steps 1-3. To help you complete steps 4-7, and come to the decision that suits you best, we have prepared a worksheet on the following page.
